
JOURNAL INFORMATION
==============================
NLM Title Abbreviation: Behav Neurol
Journal ID: Behav Neurol
NLM Journal ID: 8914585
Journal ID: BN

Behavioural Neurology

ISSN: 0953-4180
EISSN: 1875-8584
Publisher: Wiley

ARTICLE INFORMATION
==============================
PMCID: PMC5434398
PMID: 21422550
DOI: 10.3233/BEN-2010-0290
Article ID: 892373
Article version: 1
Subjects: Research Article

Neurofunctional and Neuromorphological Evidence of the Lack of Compensation in Pathological Aging

Berlingeri M. 1
Sacheli L. 1
Danelli L. 1
Ferri F. 1
Traficante D. 1
Basilico S. 2
Sberna M. 4
Sterzi R. 5
Bottini G. 2 3
Paulesu E. 6
1 Psychology Department University of Milano-Bicocca Milan Italy
2 Laboratory of Cognitive Neuropsychology Niguarda Ca’ Granda Hospital Milan Italy
3 Psychology Department University of Pavia Pavia Italy
4 Neuroradiology Department Niguarda Ca’ Granda Hospital Milano Italy
5 Neurological Science Department Niguarda Ca’ Granda Hospital Milano Italy
6 IRCSS Galeazzi Milan Italy
Print publication date: 2010
Electronic publication date: 2011 Mar 7
Volume: 23
Issue: 4
First page: 185
Last page: 187
Received 2011 Jan 31; Accepted 2011 Jan 31
Copyright: Copyright © 2010 Hindawi Publishing Corporation and the authors.
Copyright year: 2010
License: This is an open access article distributed under the Creative Commons Attribution License, which permits unrestricted use, distribution, and reproduction in any medium, provided the original work is properly cited.
License URL: https://creativecommons.org/licenses/by/3.0/

==============================
Behavioural Neurology 23 (2010) 185–187
DOI 10.3233/BEN-2010-0290
IOS Press

### Neurofunctional and neuromorphological evidence of the lack of compensation in pathological aging

M. Berlingeri[a], L. Sacheli[a], L. Danelli[a], F. Ferri[a], D. Traficante[a], S. Basilico[b], M. Sberna[d], R. Sterzi[e], G. Bottini[b,c] and E. Paulesu[f]
[a]*Psychology Department, University of Milano-Bicocca, Milan, Italy*
[b]*Laboratory of Cognitive Neuropsychology, Niguarda Ca' Granda Hospital, Milan, Italy*
[c]*Psychology Department, University of Pavia, Pavia, Italy*
[d]*Neuroradiology Department, Niguarda Ca' Granda Hospital, Milano, Italy*
[e]*Neurological Science Department, Niguarda Ca' Granda Hospital, Milano, Italy*
[f]*IRCSS Galeazzi, Milan, Italy*

### 1. Introduction

In Cognitive Neuroscience of Aging compensatory processes have been studied as age-related changes in both brain function/morphology and cognition [1]: the main trust in this research field is the idea that an aged brain can still show some plasticity or "flexible compensation", i.e. the over-recruitment of new neural networks associated with the maintenance of a successful performance [2]. This should be distinguished from other forms of "inflexible compensation" like the neurocognitive effort represented by the over-recruitment of task-specific neural networks typically activated by young healthy subjects [3]. Although these concepts have been extensively explored in *graceful aging* (the aging of normal elderly subjects), no such effort has been made for degenerative disorders yet.

In this study we investigated the neurocognitive differences between early forms of pathological (amnestic Mild Cognitive Impairment, aMCI [4]) and healthy aging in the context of compensatory processes theories. The experiment was designed to investigate the following issues: (i) are aMCIs able to recruit compensatory processes? (ii) Are aMCIs' compensatory processes qualitatively different from the ones present in healthy older subjects? (iii) Can we correlate neurofunctional inflexibility with the structural brain impairment typical of aMCIs?

### 2. Materials and methods

Twenty-four healthy elderly subjects and nine MCI patients were studied.

The neuropsychological battery included the Mini-Mental State Examination (MMSE) [5], and a series of neuropsychological tests assessing memory functions, language and executive functions.

A whole volume T1-weighted anatomical scan was acquired for each participant and processed by an optimized Voxel-Based Morphometry protocol [6].

The fMRI protocol included: picture naming (PNT), sentence judgment (SJT), picture recognition (PRT), and sentence recognition (SRT). Behavioural responses were collected during the fMRI sessions except for the picture naming where the Aachener Aphasia Test [7] performance was used as behavioural index.

The fMRI signal was pre-processed and modelled in a block design for each subject.

Then, a second-level ANOVA was estimated to calculate:

ISSN 0953-4180/10/$27.50 © 2010 – IOS Press and the authors. All rights reserved

1) Effect of pathology (under-recruitments, i.e. aMCI $<$ healthy olders; over-recruitments, i.e. aMCI $>$ healthy olders) thresholded at $p < 0.05_{uncorrected}$. An inclusive mask, corresponding to the main effect of each task at $p < 0.05_{uncorrected}$, was used to restrict each comparison to the neural network of interest.
2) Common areas not affected by pathology, assessed by a conjunction analysis of the simple effects of group in each task thresholded at $p < 0.05_{uncorrected}$ and masked in order to exclude the voxels that were significantly over- or under-recruited by aMCIs ($p < 0.05_{uncorrected}$).

To assess whether the effect of pathology fell either within the compensatory network (i.e. brain regions hyperactivated by healthy elderly subjects to maintain a good level of performance) or within the dysfunctional network (i.e. brain regions hypoactivated by healthy olders) of each task, a small volume correction (SVC) approach was used. The searching volumes corresponded to the t-maps of the hypo- and hyper-activations described in Berlingeri et al. [3] thresholded at $p < 0.01$.

Finally, the levels of grey matter (GM) density in the hippocampi and in neocortical frontal areas were correlated with the functional changes indicating a lack of flexible compensation.

### 3. Results

At the neuropsychological tests, aMCI patients showed a significant reduction of the behavioural performance only at the sort story recall [8] (Mann-Whitney U test: U = 5, Z = −3.73, $p < .001$), at the immediate and delayed recall of 15 word list [9] (U = 15.5, Z = −3.5, $p < 0.001$ and U = 14, Z = −3.59, $p < .001$ respectively) and at the delayed recall of the Rey's figure [10] (U = 32.5, Z = −3.05, $p <0 .001$).

The analyses of the behavioural responses collected during the fMRI scans revealed a significant difference between aMCIs and controls at the SJT (U = 25.5, Z = −3.48, $p < 0.001$), at the PRT (U = 26, Z = −3.33, $p < 0.001$), and at the SRT (U = 21.5, Z = −3.56, $p < 0.001$).

The VBM-analyses showed a significant atrophy in the patients' group in the medial temporal lobes, including the hippocampi and the parahippocampal gyri bilaterally ($p < 0.05$ FWE_corrected)

The fMRI results are described in Fig. 1. As expected, aMCIs showed a large number of task-specific common areas of activation with healthy controls. Yet, some task-specific differences emerged. Notably, the SVC showed that while aMCIs' over-recruitments partly overlapped with the task-specific neural networks typical of youngers (age-related dysfunctional neural network), they never included the compensatory neural networks typically activated by healthy elderly subjects, which were always under-recruited. The BOLD signals extracted from the task-specific dysfunctional neural networks were negatively correlated with the GM density in the left and in the right hippocampus for the PNT ($r = -0.44$, $p < 0.005$ and $r = -0.32$, $p < 0.04$), in the left hippocampus for the SJT ($r = -0.32$,

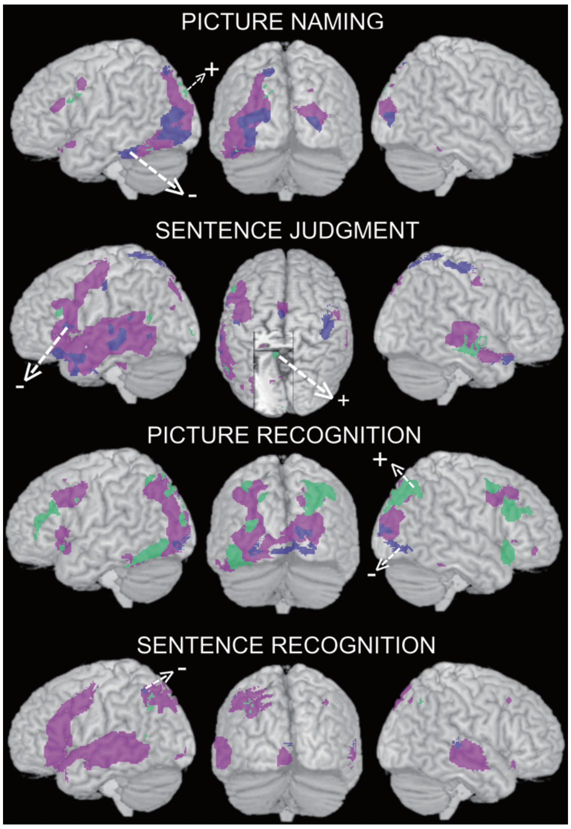

Fig. 1. fMRI results. Purple areas represent the brain regions commonly activated by aMCIs and healthy elderly controls, green areas represent aMCIs under-recruitments, while blue regions represent aMCIs over-recruitments. The symbol (+) represents the local maximum falling into the compensatory network (i.e. a network of regions representing the neurofunctional marker of compensatory processes in healthy elderly subjects challenged with the same experimental tasks) that was used for the correlation analyses. The symbol (-) stands for the local maximum falling into the dysfunctional neural network (i.e. those brain regions typically activated by youngers but no longer activated by healthy elderly subjects when challenged with the same experimental tasks) that was entered in the correlation analyses.

$p < 0.04$), PRT ($r = -0.35, p < 0.02$) and for the SRT ($r = -0.34$, $p < 0.03$). On the contrary, the BOLD extracted from the task-specific compensatory neural network showed a positive correlation with the GM density of the left hippocampus in the SJT ($r = 0.35$, $p < 0.03$), and of the left and the right hippocampus in the PRT ($r = 0.45$, $p < 0.05$, $r = 0.49$, $p < 0.05$ respectively).

### 4. Discussion

Taken together, these results suggest that our patients fail to successfully recruit alternative neural networks, and they rigidly rely on the "usual" task-specific neural networks which probably become less effective as the time goes by. The focus of our interest was the qualitative difference between the activations of healthy olders and those of aMCIs. The SVC analyses applied on the effect of pathology revealed a systematic scenario: aMCIs under-recruited areas included in the compensatory task-specific neural networks, while their over-recruitments always overlapped with parts of the neural network typically activated by youngers but not by high-performing olders, possibly a dysfunctional neural network for normal elderly. Accordingly, the coupling of behavioural and neurofunctional results emerging from the SJT, PRT and SRT can be interpreted as evidence of unsuccessful neurocognitive effort. Instead, the maintenance of a juvenile level of accuracy in the PNT associated with both the over-recruitment of the dysfunctional and the under-recruitment of the compensatory neural networks can be interpreted as inflexible compensation (or successful cognitive effort) in aMCIs.

The evidence discussed so far suggests that pathological aging could be characterized in terms of neurofunctional inflexibility. This lack of neurofunctional flexibility directly correlates with the pathology-driven GM atrophy of the hippocampi, suggesting that an underlying pathological process might be the common cause responsible for the co-occurrence of neurofunctional inflexibility and neuromorphometrical changes.

### Acknowledgements

The study was supported by the research grant RF05.103 from Ministero della Salute, Italy. The first author (MB) is supported by "Dote Riceratori" FSE, Regione Lombabrdia, Italy.

### References

[1] R. Cabeza, Cognitive neuroscience of aging: contributions of functional neuroimaging, *Scand J Psychol* **42**(3) (2001), 277–286.

[2] C.L. Grady, Cognitive neuroscience of aging, *Ann N Y Acad Sci* **1124** (2008), 127–144.

[3] M. Berlingeri et al., With time on our side? Task-dependent compensatory processes in graceful aging, *Exp Brain Res* **205**(3) (2010), 307–324.

[4] R.C. Petersen et al., Mild cognitive impairment: clinical characterization and outcome, *Arch Neurol* **56**(3) (1999), 303–308.

[5] M.F. Folstein, S.E. Folstein and P.R. McHugh, "Mini-mental state". A practical method for grading the cognitive state of patients for the clinician, *J Psychiatr Res* **12**(3) (1975), 189–198.

[6] C.D. Good et al., A voxel-based morphometric study of ageing in 465 normal adult human brains, *Neuroimage* **14** (2001), 21–36.

[7] C. Luzzatti, K. Willmes, P. Bisiacchi, R. De Bleser, L. Faglia, A. Mazzucchi et al., The Aachen Aphasia Test (AAT). II. Psychometric properties of the Italian version, *Archivio di Psicologia, Neurologia e Psichiatria* **48** (1987), 480–519.

[8] H. Spinnler and G. Tognoni, Standardizzazione e taratura italiana di test neuropsicologici. Vol. Supplementum N.8. (1987), Milano: Masson Italia Periodici.

[9] G.A. Carlesimo, C. Caltagirone and G. Gainotti, The Mental Deterioration Battery: normative data, diagnostic reliability and qualitative analyses of cognitive impairment. The Group for the Standardization of the Mental Deterioration Battery, *Eur Neurol* **36**(6) (1996), 378–384.

[10] G. Carlesimo et al., Standardizzazione di due test di memoria per uso clinico: Breve Racconto e Figura di Rey, *Nuova Rivista di Neurologia* **12** (2002), 1–13.
